# A Carbolic Acid Proving

**Published:** 1890-09

**Authors:** C. M. Selfridge

**Affiliations:** Port Townsend, State of Washington


### A CARBOLIC ACID PROVING.

EDITORS OF THE HOMŒOPATHIC PHYSICIAN:

I must tell you an experience I had with Carbolic acid about a year ago.

About half-past ten o'clock on the morning of August 7th, 1889, I was washing a Carbolic-acid bottle, and, through an accident, got some on the index finger of my left hand, over the second joint.

I went to the hydrant, turned on the water, and washed it off, as I supposed. In a minute or two I felt my finger getting numb. This numbness extended up the arm into the shoulder. Soon afterward my left leg got quite numb, so that I had some difficulty in walking. My arm felt as if deprived of all power, and I had to move it with the right hand. This numbness lasted all day until I retired at eleven o'clock P. M.

I slept well all night, and felt no inconvenience next day. But the peculiar thing about the case is that every seven days for three weeks I had a similar attack of numbness, commencing at the same time of day and lasting all day until I retired at night.

I have not tried the experiment since that time.

Believing this partial proving of Carbolic acid may be useful to some members of the profession, I communicate the facts to you.

C. M. SELFRIDGE, M. D.

PORT TOWNSEND, STATE OF WASHINGTON,
July 28th, 1890.

